# Predictive factors of continuous negative extrathoracic pressure management failure in children with moderate to severe respiratory syncytial virus infection

**DOI:** 10.1038/s41598-021-87582-4

**Published:** 2021-04-13

**Authors:** Shingo Ishimori, Yo Okizuka, Satoshi Onishi, Tadashi Shinomoto, Hirotaka Minami

**Affiliations:** 1 Department of Pediatrics, Takatsuki General Hospital, 1-3-13 Kosobe-cho, Takatsuki City, Osaka, 5691192 Japan; 2 Department of Pediatrics, Intensive Care of Medicine, Takatsuki General Hospital, Osaka, Japan

**Keywords:** Respiratory tract diseases, Risk factors, Paediatric research

## Abstract

Continuous negative extrathoracic pressure (CNEP) might be beneficial for children with severe respiratory tract infections. However, there are no available data on the predictors of its failure among individuals with respiratory syncytial virus (RSV) infections. Here, we conducted a retrospective cohort study between October 1, 2015 and October 31, 2018 in hospitalized children with moderate to severe symptoms of respiratory syncytial virus (RSV) infections. We divided 45 children requiring CNEP ventilation with a non-fluctuating negative pressure of − 12 cm H_2_O into two groups. They were classified based on improvement or deterioration of their respiratory disorder under CNEP ventilation (responder group: n = 27, failure group: n = 18). Based on the univariate analysis, the responder and failure groups significantly differed in terms of median age, days elapsed from RSV onset to the initiation of CNEP, white blood cell count (WBC), titer of venous pCO_2_, body temperature at admission, and modified Wood-Downes Score (mWDS) 6 h after initiating CNEP. Based on a logistic regression analysis adjusted for age < 1 year upon admission, less than 5 days elapsed from RSV onset to the initiation of CNEP, not high value of WBC and body temperature at admission, and high values of mWDS 6 h after initiating CNEP were found to be significant independent risk factors for CNEP ventilation failure. The former two variables were associated with less failure (odds ratio was approximately 5), and the latter two variables are associated with more failure (odds ratio was approximately 8–9). Thus, CNEP could be a valid option for children with moderate to severe RSV infections, especially in those who were aged > 1 year, and specific clinical and laboratory findings.

## Introduction

Noninvasive mechanical ventilation can support spontaneous breathing without endotracheal intubation in children with respiratory distress^1–3^. The predictive factors of noninvasive ventilation (NIV) failure in children with respiratory illness have been evaluated. Essouri et al. showed that the initial diagnosis was a critical factor for NIV efficacy, as acute respiratory distress syndrome was associated with a high risk of NIV failure^4^. Moreover, the fraction of oxygen level after 1 h of NIV^5^, decreased respiratory rate^6^, and blood gas variables^7^ could be predictors of NIV failure. Negative-pressure ventilation (NPV) is a noninvasive therapy that supports the respiratory muscles^8^. It might be effective for infants and young children with acute respiratory failure^9^. Nunez et al. reported that 69% of pediatric patients on NPV with acute respiratory failure admitted to the pediatric intensive care unit (PICU) were successfully managed^10^. In their study, compared with patients who did not respond to NPV management, those who were responsive to NPV within 1 h of initiation had a lower oxygen requirement.

Respiratory syncytial virus (RSV) is the main cause of acute viral bronchiolitis in children who may require hospital admission for lower respiratory tract disease and mechanical ventilation for progressive acute lung injury^11–13^. Acute bronchiolitis presents with fever, rhinorrhea, cough, wheezing, hypoxemia, and retractions. Infants with bronchiolitis are more likely than older children to need hospitalization^11,12^. Although the mortality of children with bronchiolitis caused by RSV infection decreased, deaths associated with RSV infection remain high^13^. A recent single-center cohort study on acute respiratory failure suggested that NPV might be effective particularly in pediatric patients with acute bronchiolitis^14^. Al-balkhi et al. have reported that endotracheal intubation may be prevented in older infants with severe respiratory tract infection who experienced apnea and who received NPV management^15^. Moreover, they showed that children receiving NPV had few complications associated with ventilation, although they often required sedation for comfort.

To date, although there are several studies on NPV management for acute respiratory failure, most used the bi-phasic mode of NPV and sedative drugs at the PICU. In addition, studies on the predictive factors of NPV included a heterogeneous group with acute respiratory failure due to any causes, including acute bronchiolitis^10,14^. Children with acute bronchiolitis who required respiratory assistance in our center have been supported with the non-bi-phasic mode of continuous negative extrathoracic pressure (CNEP), not requiring sedative drugs. As CNEP is frequently used in our site for infants with bronchiolitis, identification of risk factors for failure in RSV bronchiolitis may help identify patients for whom closer monitoring may be warranted or other modalities of support considered. In this study, we conducted a retrospective cohort study of hospitalized children with moderate to severe symptoms of RSV infections to identify the predictive factors of CNEP failure.

## Results

### Patients

**Figure 1:**
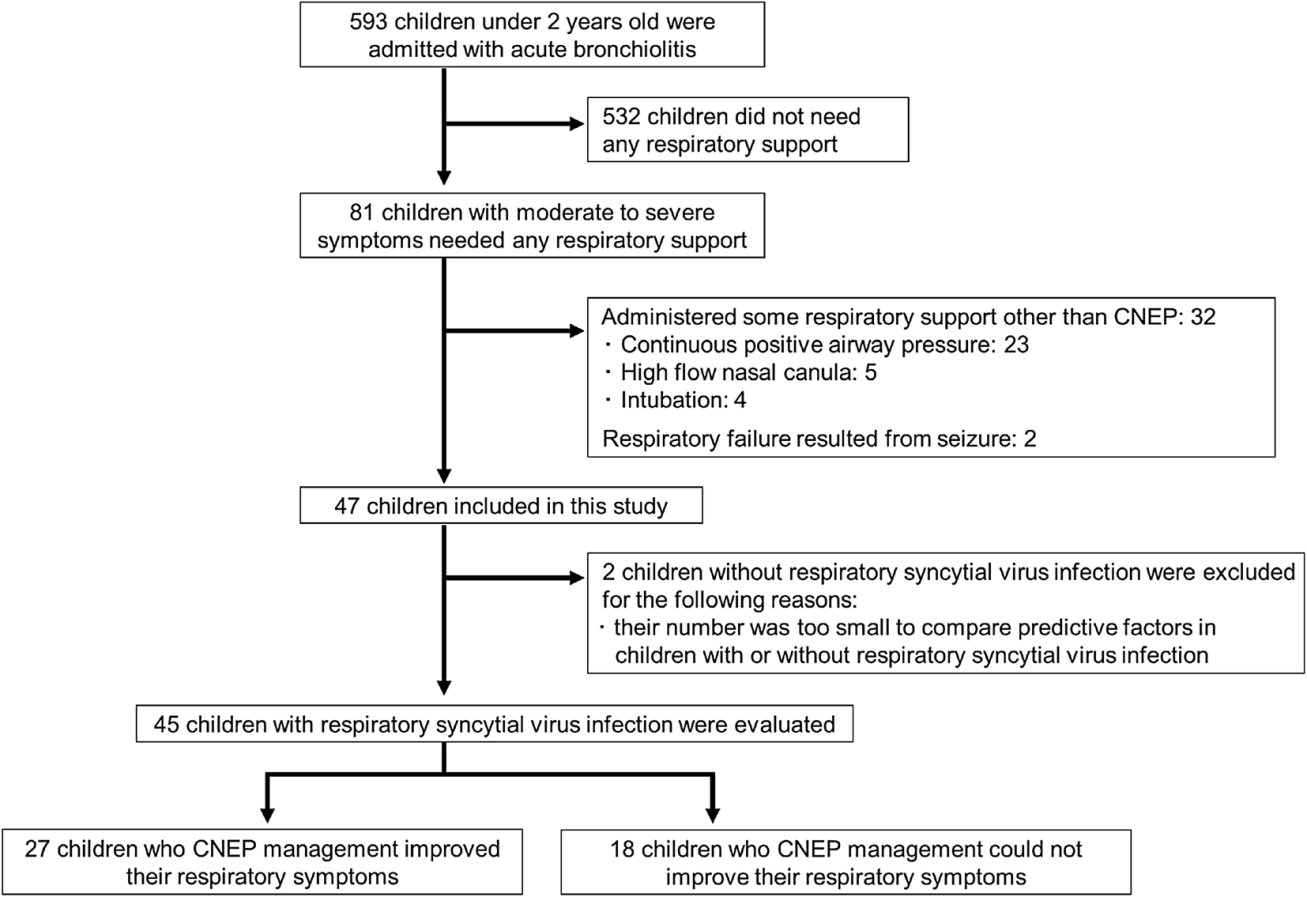
Patient flowchart. Of 593 children aged under 2 years who were admitted due to acute bronchiolitis, 45 children with moderate to severe symptoms of respiratory syncytial virus requiring respiratory support were evaluated in this study.

In total, 593 children aged under 2 years who presented with acute bronchiolitis were admitted to our hospital during the study period (Fig. 1). Of these, 81 children with moderate to severe symptoms received some type of respiratory support. Thirty-two children were excluded because they received treatment with other respiratory devices, 23 who received nasal or mask continuous positive airway pressure (CPAP), 5 who received nasal high-flow therapy, and 4 who required immediate intubations. Two children with respiratory failure from convulsions were also excluded. Because only two children had non-RSV bronchiolitis, we restricted our analysis to only those with RSV. Therefore, 45 children with RSV infection were finally include in this study. The median age at admission was 0.8 years. The median age at admission in the failure group was significantly lower than that in the responder group (Table 1). Median gestational age, birth weight, and proportion of patients with other past history conditions were not significantly different between the responder and the failure groups. The details of past medical history, except for premature birth and low birth weight, were as follows: two children presented with recurrent wheezing, one with trisomy 21, one with peripheral pulmonary stenosis, and one with arachnoid cysts.

**Table 1:** Background between responder and failure group.

	All (n = 45)	Responder group (n = 27)	Failure group (n = 18)	*P* value
Male : female	27:18	16:11	11:7	> 0.99
Age at admission (years old)	0.8 (0.3–1.3)	1 (0.7–1.3)	0.3 (0.1–0.8)	< 0.001
Gestational week (weeks)*	39 (37–40)	39 (37–40)	39 (37.5–39.5)	0.90
Birth weight (g)^†^	2985 (2579–3302)	2987 (2439–3331)	2985 (2684–3322)	0.58
Past history except for prematurity and low birth weight ^‡^	5/37 (14%)	2/24 (8%)	3/13 (13%)	0.32

Values are median + interquartile ranges, *; Evaluated 37 children who could collected the data of Gestational week, †; Evaluated 38 children who could collected the data of Birth weight, ‡; Evaluated 37 children who could collected the data of Gestational week and Birth weight.

### Characteristics and clinical course during admission (univariate analysis)

Table 2 shows the comparison of the patients’ characteristics between the responder and the failure groups. At admission, no statistically significant differences between the groups for clinical characteristics and modified Wood-Downes Score (mWDS)^16^ values were found. The median body temperature upon admission differed significantly between the responder and failure groups. Laboratory data upon admission in the failure group revealed that the median white blood cell (WBC) count was significantly lower and the median pCO_2_, HCO_3_, and base excess levels of venous blood gas were significantly higher than those in the responder group. The median number of days elapsed from RSV onset to the initiation of CNEP ventilation was significantly shorter in the failure group than in the responder group (5.0 vs 6.0 days). The mWDS values at the time of starting CNEP support and the difference between mWDS values at the time of admission and at the time of starting CNEP ventilation did not differ significantly between the responder and the failure groups. Children in the failure group exhibited significantly greater mWDS values at 6 h after initiating CNEP ventilation and significantly smaller differences between mWDS values at the start of CNEP ventilation and at 6 h after initiating CNEP ventilation. Similar proportions of patients in each group used oxygen, antibiotics, general steroids, and any inhaler (Table 3). Median total durations of admission and oxygen use were significantly greater in the failure group compared with those in the responder group. All CNEP failure children were transferred to the PICU for another type of ventilation with sedation. In the failure group, only one child had his respiratory condition deteriorate despite CPAP ventilation with sedation in the PICU and required endotracheal intubation. No children presented with a skin injury or hypothermia under CNEP management. Three children (11%) in the responder group had sedative drugs during admission. However, they required sedation due to their turbulence for being hospitalized. In addition, of three children transferred to the PICU, one required sedation due to her turbulence for being hospitalized. She started sedation at the PICU before initiating CNEP management. Two children were transferred to the PICU for intensive sputum therapy before initiating CNEP management without sedation. Although these three children received CNEP at the PICU, they were not defined as CNEP failure.

**Table 2:** Comparison of characteristics between responder and failure group.

	All (n = 45)	Responder group (n = 27)	Failure group (n = 18)	*P* value
**Characteristics at admission**				
Elapsed days from RSV onset (days)	5.0 (3.5–6.0)	5.0 (3.0–7.0)	4.0 (3.8–5.0)	0.08
Respiratory rate (times/min)	46.5 (40–57.5)	45 (40–60)	48 (35–55)	0.97
Heart rate (times/min)	160 (150–170.5)	160 (151–170)	160 (148.5–180)	0.81
Body temperature (℃)	38.6 (37.6–39.7)	38.7 (38.4–39.8)	37.8 (37.4–39.1)	0.03
mWDS	2.0 (1.0–3.8)	2.0 (1.0–3.5)	2.0 (1.0–4.0)	0.85
**Laboratory data at admission**				
White blood cell (/μl)	10,900 (8400–13,300)	12,200 (10,000–14,100)	9100 (6325–11,000)	0.003
Venous pH	7.37 (7.33–7.40)	7.38 (7.34–7.41)	7.34 (7.32–7.39)	0.14
Venous pCO_2_ (mmHg)	38.6 (34.3–43.9)	37.5 (32.3–40.8)	45.5 (35.7–52.1)	0.02
Venous HCO_3_ (mmHg)	21.3 (20.2–23.7)	21.0 (20.0–22.0)	24.0 (20.4–26.8)	0.02
Venous Base excess	−3.1 (−3.9–−1.5)	−3.3 (−4.2–−2.0)	−2.0 (−3.4–−0.5)	0.04
Venous lactate (mg/dl)	15.0 (12.3–18.0)	15.5 (12.3–19.8)	14.5 (12.5–16.0)	0.44
**Characteristics at initiating CNEP**				
Elapsed days from RSV onset (days)	5.0 (4.0–7.0)	6.0 (5.0–7.0)	5.0 (4.0–5.3)	0.02
Respiratory rate (times/min)	50 (41–60)	40 (50–60)	60 (46.5–60)	0.10
Heart rate (times/min)	150 (134–172)	145 (130–162)	158 (145–183)	0.06
Body temperature (℃)	37.9 (37.0–40.1)	37.7 (37.0 to 38.7)	37.9 (37.2–39.2)	0.49
Times from admission to initiating CNEP (hours)	11 (0–24)	14 (4–29)	8 (0–15)	0.12
mWDS	4.0 (3.5–5.0)	3.5 (4.0–5.0)	4.0 (3.9–5.0)	0.53
The mWDS values differences between at the time of admission and initiating CNEP	2.0 (1.0–3.5)	2.0 (1.0–3.0)	3.0 (1.0–3.5)	0.46
**Characteristics 6 h after initiating CNEP**				
mWDS	3.5 (2.0–4.0)	2.5 (2.0–3.5)	4.0 (3.4–4.6)	< 0.001
The mWDS values differences between at the time of initiating CNEP and 6 h after initiating CNEP	−1.0 (−2.0–0)	−1.0 (−2.5–−0.5)	0 (−1.0–0.1)	0.001

*RSV* respiratory syncytial virus, *CNEP* continuous negative extrathoracic pressure, *mWDS* modified Wood-Downes Score, Values are median + interquartile ranges.

**Table 3:** Comparison of clinical course between responder and failure group.

	All (n = 45)	Responder group (n = 27)	Failure group (n = 18)	*P* value
**Clinical course during admission**				
Total duration of CNEP management (days)	2 (2–3.5)	3 (2–4)	1.5 (1–2.3)	0.003
Total duration of admission (days)	8 (6–10)	7 (6–8)	11 (9.5–12)	< 0.001
Transfer to PICU	21 (47%)	3 (11%)	18 (100%)	< 0.001
Total PICU length of stay (days)	6 (4.8–8)	3 (2–4)	7 (5–8)	0.012
Use of sedative drugs	21 (47%)	3 (11%)	18 (100%)	< 0.001
Use of oxygen	45 (100%)	27 (100%)	18 (100%)	> 0.99
Total duration of oxygen (days)	7 (5–8)	5 (3–7)	8.5 (7.3–10.8)	< 0.001
Use of antibiotics	21 (47%)	15 (56%)	6 (33%)	0.22
Total duration of antibiotics (days)	5 (4–6)	5 (4–6)	5 (4.5–6)	0.86
Use of general steroid	5 (11%)	3 (11%)	2 (11%)	> 0.99
Use of any inhaler	21 (47%)	12 (44%)	9 (50%)	0.77
Salbutamol	12 (27%)	8 (30%)	4 (22%)	0.74
Epinephrine	3 (7%)	2 (7%)	1 (6%)	> 0.99
Other	6 (13%)	2 (7%)	4 (22%)	0.2

*CNEP* continuous negative extrathoracic pressure, *PICU* pediatric intensive care unit, Values are median + interquartile ranges.

### Predictive factors of CNEP management failure (multivariate analysis)

Based on a logistic regression analysis adjusted for age < 1 year upon admission, less than 5 days elapsed from RSV onset to the initiation of CNEP, high WBC count and hyperthermia upon admission, and high clinical index 6 h after initiating CNEP were found to be the significant independent risk factors associated with CNEP ventilation failure (Table 4). The former two variables were associated with less failure (odds ratio was approximately 5), and latter two variables are associated with more failure (odds ratio was approximately 8–9).

**Table 4:** Adjusted for variables including age < 1 or ≥ 1 year at admission and (a) less than 5 days elapsed from RSV onset to the initiation of CNEP or more than 5 days, (b) WBC count < 10,000 or ≥ 10,000 /µl, (c) venous blood pCO2 levels ≥ 40 or < 40 mmHg at admission, (d) body temperature < 38 ℃ or ≥ 38 ℃ at admission, (e) mWDS ≥ 4 or < 4, 6 h after initiating CNEP.

	Odds ratio	95% confidential interval	*P* value
**(a)**			
The age at admission < 1 years old (reference: ≥ 1 years old)	8.4	1.73–66.6	0.007
Days elapsed from RSV onset to initiation of CNEP < 5 (reference: ≥ 5)	5.6	1.27–31.3	0.02
**(b)**			
The age at admission < 1 years old (reference: ≥ 1 years old)	9.1	1.80–76.1	0.006
White blood cell < 10,000/µl (reference: ≥ 10,000 /µl)	5.6	1.28–30.3	0.02
**(c)**			
The age at admission < 1 years old (reference: ≥ 1 years old)	13.6	2.14–267.3	0.004
Venous pCO2 ≥ 40 mmHg (reference: < 40 mmHg)	1.74	0.39–7.96	0.46
**(d)**			
The age at admission < 1 years old (reference: ≥ 1 years old)	7.8	1.50–64.2	0.012
Body temperature < 38 ℃ (reference: ≥ 38 ℃)	8.3	1.90–45.8	0.004
**(e)**			
The age at admission < 1 years old (reference: ≥ 1 years old)	8.8	1.70–75.8	0.009
mWDS ≥ 4, 6 h after initiating CNEP (reference: < 4)	9.0	2.10–49.4	0.003

## Discussion

To the best of our knowledge, this is the first study to identify the factors associated with CNEP failure in children with RSV infection. We showed that CNEP management could be a valid option for some children with moderate to severe RSV infections.

In this study, we determined predictive factors associated with failure of CNEP management in children with moderate to severe RSV infection. Children younger than 1 year old exhibited a significantly greater risk of CNEP support failure, similar to the findings in infants with severe RSV infection^7,17^. Previous studies revealed factors related to CPAP failure in children with RSV infection and bronchiolitis. In a prospective study of 101 children with severe RSV infection^7^, the predictors of failure were apnea, high pCO_2_ upon admission, and the Pediatric Risk of Mortality (PRISM) score at 24 h after initiating CPAP, which was the physiology-based score for mortality risk^18^. Larrar et al.^19^ determined that the predictive factors of nasal CPAP failure were only the PRISM score calculated at day 1 and the initial reduction of pCO_2_ in 145 infants with severe acute bronchiolitis. In our population, univariate analysis showed the pCO_2_ upon admission was significantly higher in the failure group than in the responder group. However, multivariate analysis revealed no significant difference. Similar to studies that investigated the predictive factors of NIV failure^5–7^, our research revealed that non-improvement in respiratory symptoms within the first 6 h is a factor for CNEP support failure. High WBC counts at admission in the responder group seemed to be complications of secondary bacterial infection; however, the proportion of patients treated with antibiotics was not significantly different between the two groups. Age at admission and days elapsed from the onset of RSV infection could have affected WBC count. In a prospective study of 565 hospitalized children less than three years old with RSV infection, secondary bacterial infection was uncommon (developed in only 1.2 percent)^20^. Half of our patients required antibiotics for subsequent bacterial infection. We suppose that as all children in the present study required respiratory mechanical ventilation, that the risk of secondary bacterial infection was increased as with the studies in children who were admitted to the intensive care unit^21,22^.

A recent cohort study, evaluated the predictive factors of NPV in children with all-cause respiratory failure and showed that the proportion of acute bronchiolitis was significantly higher in NPV responders^14^. In general, RSV is the most common cause of bronchiolitis, which is a significant cause of hospitalization in infants and young children^23–25^. Although identification of risk factors for failure in attention to the clinical course of acute bronchiolitis may help identify children who will be warned of close surveillance, there are no published data. The predictive factors of NIV focusing on days elapsed from the onset of RSV infection have not been evaluated. The typical clinical course of bronchiolitis begins with nasal congestion or discharge, followed by wheezing or crackles^26–28^. Although respiratory findings of acute viral bronchiolitis vary substantially over time and the mean duration is around two weeks, clinical symptoms would gradually resolve after the peak three to five days after illness onset. Our multivariate analysis indicated that if children with moderate to severe RSV require any respiratory support after 5 days from disease onset, their respiratory disorder could improve under CNEP ventilation because the peak RSV activity was already past.We should consider the pediatric indication of CNEP support, considering the number of days elapsed from the onset of RSV infection. Of clinical scoring, the increase in mWDS between admission and initiating NPV in our population can be attributed to two causes. First, few children had severe condition requiring respiratory support upon admission. Second, the peak RSV activity in children receiving NPV management have not passed yet at the time of admission.

The NPV ventilation setting in our study was different from that in previous reports. That is, we treated children with acute bronchiolitis with a continuous negative pressure of −12 cm H_2_O and not the bi-phasic mode that has been previously described. A recent study in 233 pediatric subjects with acute respiratory failure revealed response rate and limitation of NPV with the bi-phasic mode^14^. Bi-phasic mode of NPV is controlled in synchronization with spontaneous breathing, which necessitates intravenous sedation in patients. There was a delay in enteral nutrition caused by constant sedation^14^. Most of our children did not require sedative drugs for CNEP ventilation.

Several limitations to this study exist. First, it was a retrospective chart research in a single center and did not have a control group. A randomized, controlled trial in multiple centers is required to evaluate the clinical efficacy, and failure of CNEP ventilation. A second limitation of the present study is that our tertiary hospital, where children with severe respiratory distress are often referred, may be associated with selection bias. Being careful in applying these results is necessary, as not all children with RSV infection need respiratory ventilation. Third, we did not examine the efficacy of CNEP support except in those with respiratory distress from RSV infection.

In conclusion, CNEP could be a valid option for some children with moderate to severe RSV infections. Days after illness onset, age, clinical and laboratory findings upon admission, and respiratory score after initiating respiratory support might be the predictors of CNEP failure in pediatric RSV infections.

## Methods

### Subjects and study design

We retrospectively investigated the records of children with acute bronchiolitis who were admitted to our hospital between October 1, 2015, and October 31, 2018, when CNEP therapy was available for children and complete data on demographic and clinical characteristics and course during hospitalization were obtained. We included children who were managed with CNEP support and were aged <2 years at the time of admission. We excluded children with chronic lung disease or those who used respiratory devices at home, which included oxygen therapy, home ventilators, and tracheostomy management. Other reasons for exclusion were if children needed immediate endotracheal intubation or exhibited contraindications for CNEP ventilation, including upper airway obstruction, thoracic trauma, pneumothorax, hemothorax, tracheomalacia, or central alveolar hypoventilation syndrome. In eligible patients, the following variables were evaluated: sex, age at admission, gestational weeks, birth weight, previous medical history other than prematurity or low birth weight, presence of RSV infection, use of oxygen, duration of oxygen therapy, use of general steroids and any inhaler, duration of hospital stay, and laboratory data upon admission, including WBC count, venous pH, pCO_2_, HCO_3_, base excess, and lactate levels. In addition, we collected clinical data at admission and also at the initiation of CNEP ventilation, including the days elapsed from the onset of RSV infection, body temperature, respiratory rate, heart rate, and mWDS. This study was conducted in accordance with the Declaration of Helsinki and with the ethical guidelines for Medical and Health Research Involving Human Subjects promulgated by the Ministry of Health, Labor and Welfare in Japan. The institutional ethics review board of Takatsuki General Hospital approved this study (approval number: 2018-87). This ethics committee clearly stated that the researchers did not need to obtain informed consent, because complete data in this study were collected from patient medical records. According to this statement, informed consent was not obtained from patients or their parents. In accordance with the guidelines and the institutional ethics review board for the patients’ benefit, the protocol was displayed publicly in a poster at Takatsuki General Hospital, so that each patient could have opportunities to refuse to attend this study.

### Definitions

RSV infection was diagnosed using rapid antigen detection tests of a nasopharyngeal swab. The day when children first developed either a clinical symptom (coryza or cough or fever) or an abnormal physical examination sign (wheeze or crackle) indicative of RSV infection was defined as day 1. The mWDS is an index of clinical respiratory disorders that includes transcutaneous oxygen saturation (SpO_2_), inspiratory breath sounds, expiratory wheezing, respiratory muscle work load, and cerebral function^16^.

### Treatment and monitoring protocols

At our institution, children with acute bronchiolitis received oxygen therapy, not nasal high-flow therapy, to maintain SpO_2_ levels at >95%. All children underwent infusion therapy to correct dehydration and intranasal suction therapy. Some children subsequently used an inhaler to promote sputum discharge (salbutamol, bromhexine hydrochloride, epinephrine, and 3 % hypertonic saline). If patients over 6 months old exhibited discomfort and a temperature >38°C, acetaminophen was given as an antipyretic. Children who exhibited asthmatic attacks were treated with injected general steroids and an inhaled bronchodilator. In our center, when children with acute bronchiolitis required respiratory support, CNEP ventilation is initially administered. CNEP support was managed with an RTX respirator (Medivent Ltd., London, UK) and with a set negative-pressure of −12 cm H_2_O. We used three different sizes of cuirass: 0 (neonatal), 1 (infant), and 2 (toddler); the cuirass was selected to fit each child. As a general protocol in our center, sedation is not required when CNEP ventilation is administered. However, it was allowed. Patients with CNEP ventilation were monitored by electrocardiography and a pulse oximeter, and oxygen was used to maintain SpO_2_ levels >95%. After initiating CNEP support, we evaluated respiratory findings (inspiratory and expiratory respiratory sounds, types of breathing efforts), body temperature, respiratory and heart rate with cuirass performed by a pediatric center nurse or physical therapist, at least every few hours within the first 6 h and every 6 h thereafter. Skin injury, interference with access, discomfort from fitting a cuirass, and neck excoriation were evaluated with the cuirass off by a pediatric center nurse at least every few hours. We selected other respiratory support (NIV or high-flow nasal cannula) and not CNEP in children who would be prone to hypothermia, skin injury, and neck excoriation, which are recognized as complications of CNEP ventilation. We did not select the bi-phasic mode of NPV using cuirass as noninvasive respiratory ventilation because children initially treated with this mode required synchronized spontaneous breathing and sedative drugs. CNEP failure was defined when children under CNEP ventilation showed respiratory symptoms requiring immediate intubation, severe chest wall retraction or nasal alar breathing or grunting even under at least several hours of CNEP management, and/or SpO_2_ levels <90% despite using 10 L/min oxygen even under at least several hours of CNEP management. According to these definitions, children were assigned to either the responder or failure groups. If the patients’ chest wall retraction or nasal alar breathing improved under CNEP management, the setting was then weaned in steps by subtracting one or two sets and stopped as soon as possible. If improvement of their respiratory symptoms could lead to discontinue CNEP ventilation, they were considered a CNEP responder. Following discontinuation of CNEP ventilation, some children started nasal high-flow therapy and others just had inspired oxygen. Any recurrence of clinical symptoms requiring respiratory support led to reinstitution of CNEP management. We did not use mWDS values and laboratory data (including WBC count, venous pH, pCO_2_, HCO_3_, base excess, and lactate) when defining failure of CNEP management because we calculated mWDS values not on treating patients but by investigating the clinical data of medical records retrospectively. If CNEP ventilation could not improve chest wall retraction or nasal alar breathing, patients were transferred to the PICU, and they underwent intubation or received CPAP treatment with sedation. CPAP was delivered using a nasal prong or face mask. Midazolam and dexmedetomidine combined with fentanyl were used as sedative drugs in the PICU. Patients were considered for immediate endotracheal intubation as follows: (1) if they did not breathe spontaneously, (2) they had mandibular breathing or agonal respiration, (3) they had venous blood pCO_2_ levels of >70 mmHg, (4) they had SpO_2_ levels of <90%, despite using 10 L of inspired nebulized oxygen, or (5) they were unconscious due to their respiratory failure.

### Statistical analysis

All statistical analyses were performed using JMP version 11.0 (SAS institute Japan Ltd., Tokyo, Japan). We used the non-parametric method for continuous variables and the Fisher’s exact test for categorical values. All data were expressed as median value + interquartile ranges. A *P* value < 0.05 was considered statistically significant. The risk ratio for failure of CNEP management was calculated with 95% confidence intervals (CIs). Odds ratios and their 95% CIs for failure of CNEP ventilation were estimated by logistic regression model. This regression was adjusted for variables including age < 1 or ≥ 1 year upon admission, less than 5 days elapsed from RSV onset to the initiation of CNEP or more than 5 days, body temperature of < 38°Cor ≥ 38 °C upon admission, WBC count of < 10,000 or ≥ 10,000 /μL upon admission, venous blood pCO_2_ levels of ≥ 40 or < 40 mmHg upon admission, mWDS of < 4 or ≥ 4 6 h after initiating CNEP. These were variables were found to be significantly associated with CNEP failure in the univariate analysis. We performed several logistic regression analyses adjusting for just two variables. The first variable was age at admission as background, and the other was laboratory data or days elapsed from RSV as factors during or after admission. Venous blood HCO_3_ levels upon admission, venous base excess upon admission, the mWDS values and the mWDS differences between at the time of initiating CNEP and 6 h after initiating CNEP were excluded from the multivariate analysis because these factors were associated with those previously mentioned.
